# Fitness Cost Implications of PhiC31-Mediated Site-Specific Integrations in Target-Site Strains of the Mexican Fruit Fly, *Anastrepha ludens* (Diptera: Tephritidae)

**DOI:** 10.1371/journal.pone.0109690

**Published:** 2014-10-10

**Authors:** José S. Meza, Francisco Díaz-Fleischer, Lázaro R. Sánchez-Velásquez, Cristina Silvia Zepeda-Cisneros, Alfred M. Handler, Marc F. Schetelig

**Affiliations:** 1 Programa Moscafrut, SAGARPA-IICA, Metapa de Domínguez, Chiapas, México; 2 Instituto de Biotecnología y Ecología Aplicada (INBIOTECA), Universidad Veracruzana, Xalapa, Veracruz, México; 3 Center for Medical, Agricultural, and Veterinary Entomology, Agricultural Research Service, U.S. Department of Agriculture, Gainesville, Florida, United States of America; 4 Justus-Liebig-University Giessen, Institute for Phytopathology and Applied Zoology, Giessen, Germany; Virginia Tech, United States of America

## Abstract

Site-specific recombination technologies are powerful new tools for the manipulation of genomic DNA in insects that can improve transgenesis strategies such as targeting transgene insertions, allowing transgene cassette exchange and DNA mobilization for transgene stabilization. However, understanding the fitness cost implications of these manipulations for transgenic strain applications is critical. In this study independent *piggyBac*-mediated *attP* target-sites marked with DsRed were created in several genomic positions in the Mexican fruit fly, *Anastrepha ludens*. Two of these strains, one having an autosomal (*attP*_F7) and the other a Y-linked (*attP*_2-M6y) integration, exhibited fitness parameters (dynamic demography and sexual competitiveness) similar to wild type flies. These strains were thus selected for targeted insertion using, for the first time in mexfly, the *phiC31*-integrase recombination system to insert an additional EGFP-marked transgene to determine its effect on host strain fitness. Fitness tests showed that the integration event in the *int*_2-M6y recombinant strain had no significant effect, while the *int*_F7 recombinant strain exhibited significantly lower fitness relative to the original *attP*_F7 target-site host strain. These results indicate that while targeted transgene integrations can be achieved without an additional fitness cost, at some genomic positions insertion of additional DNA into a previously integrated transgene can have a significant negative effect. Thus, for targeted transgene insertions fitness costs must be evaluated both previous to and subsequent to new site-specific insertions in the target-site strain.

## Introduction

Germ-line transformation has been used widely to create genetically modified insects (GMI) in pest species, and is based primarily on vectors that are Class II transposable elements such as *piggyBac, Mariner, Hermes*, or *Minos* [1]–[4]. Transposon-mediated transformation has been used to create a variety of GMIs having the potential to increase the efficiency of insect control programs, especially those that use the sterile insect technique (SIT) [5]. These GMIs include those, most simply, having fluorescent protein transformation markers that can also facilitate the identification of sterile adults released into the wild [6]. Some strains have specifically marked tissues, such as sperm, to distinguish gender [7] and to detect mated females in the field [8], [9]. Transgenic female-specific conditional lethality strains have been created that may find use for sexing in rearing [10], [11], and non-sex-specific lethality strains may provide a means for genetic sterility that eliminates the need for male irradiation [12]. Given that the SIT control strategy bases its efficacy on the mating competition of released males with wild males, alternatives to the debilitating effects of irradiation is a high priority.

*piggyBac* transformations in the Mexican fruit fly (mexfly), *Anastrepha ludens*, have been achieved that provide strains for improved population control with the possibility of stabilizing the transgene vector by post-integration deletion of its terminal sequences [13], [14] or adding tissue-specific marking [9], [15]. While these and additional transgenic strains were created relatively efficiently in tephritid species, a limitation of transposon-mediated transformation is the random nature of their genomic integrations. This often results in insertional mutations that can diminish host strain viability and fitness [16], and unreliable transgene expression, if not silencing, due to genomic position effects [17], [18]. Both of these limitations have a particularly negative effect on the creation of transgenic strains for applied purposes that, generally, must be highly fit and exhibit optimal transgene expression. Thus, transgenic strain development typically requires creation of 10 or more independent strains with each subjected to rigorous scrutiny to select an optimal GMI for potential use in control programs.

To ameliorate the limitations of random genomic integration, new *piggyBac* transformation vectors have been developed that provide recombination acceptor sites for targeted integrations. Lines created with these vectors can be selected for strong transgene marker expression and minimal fitness effects, and then used for subsequent targeted transgene insertions. Recombinase-based targeting systems include FLP-*FRT* from *Saccharomyces cerevisiae* [19] and CRE-*loxP* from bacteriophage P1 [20], which have been used for recombinase-mediated cassette exchange in *Drosophila* and the tephritid fly, *Anastrepha suspensa* [21], [22]. The most widely used targeting system, however, is the *phiC31* integrase-based system from the bacteriophage of *Streptomyces* [23], [24].

The *phiC31* integrase normally catalyzes recombination between the bacteriophage *attP* site and the *attB* site present in the host bacterial genome [24] and has been functional in plants and animals [23], [25]–[27]. In insects, *attB/attP* recombination was first demonstrated in *Drosophila melanogaster* [28] with the first non-drosophilid recombination achieved in *Aedes aegypti* [29], followed by two other mosquito species [30]. The *phiC31* system has also been used for targeting in the Mediterranean fruit fly, *Ceratitis capitata*, and notably, it was used to introduce sequences that allowed the post-integration stabilization of the *piggyBac* vector target site [18].

While the successful use of genomic targeting systems is encouraging, it is important to know if the successive integration of transgenes into an otherwise innocuous target site can have a negative effect on transgene expression or on insect fitness. Fitness has two main components, viability through life stages and reproduction, and can be determined by analyzing demographic and behavioral parameters, such as fecundity, fertility, developmental rate, adult emergence, male ratio, and mating competitiveness [31]. Many studies have demonstrated that transgenic insects exhibit fitness costs when comparing them with their wild type counterparts [32], [33]. In SIT programs, reduced fitness of released transgenic males is likely to reduce their mating competitiveness, thus requiring more frequent releases at higher release ratios [34]–[36]. Therefore, before releasing a transgenic insect it necessary to confirm its ability to compete successfully with the indigenous population.

Here we conducted a comparative fitness analysis of different transgenic target-site strains (TTSS), created by the random integration of a *piggyBac* vector, carrying the *phiC31 attP* target-site, at different genomic positions in *A. ludens*. In a second phase of the study, two TTSS having relatively robust transgene marker expression and fitness parameters relative to wild type, were selected for *phiC31*-mediated site-specific integration of a new transgene. An evaluation of the fitness parameters and marker gene expression of the recombinant strain compared to the host strain provides insights into the possible effects of successive site-specific integrations. While the term ‘fitness’ can include a large number of physiological and behavioral parameters, herein it is referred to the most critical life-cycle and competitiveness parameters relevant to mass rearing and mating ability of released males for the sterile insect technique.

## Materials and Methods

### Insect strains

All TTSS were derived from the *Anastrepha ludens* Moscafrut colony, which has been maintained in a breeding program since 1992 in the Moscafrut bio-complex near Tapachula, Chiapas, Mexico. The *A. ludens* Chiapas colony is another strain obtained from a collection of infested fruits (sour orange, *Citrus aurantium* L. and mango, *Mangifera indica* L.) in highland and lowland regions of the state of Chiapas (sampling sites with GPS coordinates: Motozintla, 15.3167/−92.3333; Mazapa de Madero 15.40/−92.1667; La trinitaria, 16.1263/−92.027; Comitan, 16.25/−92.1333; Tzitmol, 16.27/−92.27; Tapachula, 14.9/−92.2833; Huixtla, 15.15/−92.4667; Mapastepec, 15.433/−92.9). No permissions were required for sampling the fruits and no endangered species were sampled. This strain has been inbred for 25 generations in the genetic sexing laboratories of the Moscafrut facility and was used as wild type control (WT). Both wild and transgenic strains were maintained on an artificial larval diet [37], under environmental conditions of 25°C, at 70–80% RH under a photoperiod of 12∶12 h of light∶darkness. Wild *A. ludens* females were used for sexual competitiveness tests. These were extracted as third instar larvae from sour oranges, *Citrus aurantium* L., collected in the Soconusco region of Chiapas, Mexico.

### Transformation vectors

The *piggyBac* transposon vector pBXLII*[attP-PUbDsRed_fa]* (#1425) [11] and the helper vector *phspBac* [38] were described previously. The pSL*[3pB_attB285_PUbEGFP]* donor vector for site-specific integration was generated by ligating the *Afl*II/*Spe*I cut vector backbone of pSL*[3pB_attB285_PUbDsRed]* to the *Afl*II/*Spe*I PCR fragment of *PUbnlsEGFP* amplified from pB[*PUbnlsEGFP*] [38]. The vector pSL*[3pB_attB285_PUbDsRed]* was generated by ligating an *Apa*I/*Sac*I cut *attB285* fragment from pTA*attB* [39] into *Apa*I/*Sac*I digested pSL*af_3pB-attB-PUbDsRed_af* [18].

The integrase helper vector *phs-dphiC31* was constructed by recombining the *Eco*RV cut *phspBac* vector backbone and the *dphiC31* integrase PCR fragment amplified by primer pair P819 and P820 on p*3xP3EGFP-vas-dphiC31attB* [40], and the *pBac 3′UTR* fragment amplified by primer pair P817 and P818 on *phspBac* using the GeneArt Seamless Cloning kit (Invitrogen) (Table S1 in File S1).

### Creation of transgenic target-site strains

Embryonic microinjection for germ-line transformation in *A. ludens* was carried out as described [6], [15]. To introduce *attP* target-sites into the Moscafrut wild-type strain, the vector *pBXLII[attP_PUbDsRed]* was co-precipitated with the helper plasmid *phspBac*, resuspended in injection buffer (5 mM KCl; 0.1 mM sodium phosphate pH 6.8) at a vector∶helper concentration of 600∶400 ng/µl, and micro-injected into G_0_ embryos. Surviving G_0_ adults were separated by sex and mated in small groups (families) of three G_0_ individuals to four wild-type Chiapas mexflies of the opposite sex. G_1_ progeny were screened by epifluorescence microscopy to select insects exhibiting DsRed fluorescence using a Leica TXR filter (ex: 560/40; em: 610 LP). From each transformant family a fluorescent G_1_ male was backcrossed to four wild-type Chiapas females. TTSS having single vector integration were selected by Mendelian segregation of the DsRed marker in G_2_ progeny, which were inbred to generate homozygous strains.

#### TTSS analysis

To determine independent genomic integrations of target site vectors, genomic DNA from each line was isolated using DNAzol with genomic insertion site DNA flanking each vector insert amplified by Thermal Asymmetric Interlaced (TAIL)-PCR. PCR conditions used were described by Liu and Whittier [41] with specific oligos designed to the 5′ and 3′ pBac ends being L1-5pB, L2-5pB, L3-5pB and R1-3pB, R2-3pB, R3-3pB (Table S1 in File S1). Successful PCR products were resolved by 1% agarose gel electrophoresis to verify amplified fragment sizes, subcloned in pCR4 and sequenced (Macrogen USA).

### Site-specific integration into target-site strains

The donor vector plasmid, *pSL_3pB-attB-PUbEGFP*, and the integrase helper plasmid, *phs-dphiC31*, were co-injected at a donor∶helper concentration of 250∶150 ng/µl, into embryos from TTSS selected for site-specific insertion. Virgin G_0_ adults were backcrossed in single pairs to wild type Chiapas insects, with G_1_ adults screened by epifluorescence microscopy for DsRed (TXR filter) and EGFP (Leica YFP filter; ex: 510/20, em: 560/40) fluorescence. Homozygous and heterozygous site-specific integration strains (SSIS) were distinguished by fluorescence intensity that allowed the generation of homozygous strains.

#### Insertion-site verification

To molecularly verify *attP/attB* site-specific integrations in each SSIS, the recombined *attL* sequence was amplified from genomic DNA by PCR using the primer pair JS01_R and JS01_F (Table S1 in File S1).

### Demographic parameters and sexual competitiveness

Biological fitness was estimated by an analysis of dynamic demography (life cycle transitions and population growth rates) and the level of sexual competitiveness of transgenic males in relation to WT males when mated together with wild females. These analyses were performed in two phases: one conducted for the TTSS strains and the WT strain to select TTSS that were not significantly different; and a second analysis comparing the site-specific integration strain (SSIS) to the original TTSS, in addition to the WT strain.

#### Demographic parameters

Twenty transgenic homozygote pairs of each transgenic strain and the WT strain were placed separately in 4L plastic containers adapted as cages with food and water (1∶3 protein and sugar mixture). To estimate survival from one developmental stage to the next, 100 newly oviposited eggs per line were placed on black cloth and moist filter paper within a Petri dish. These were incubated at 26°C, 70–80% RH for three days (one day previous to hatching) at which time the black cloth with eggs was transferred to new dish with artificial diet. On the third day with diet the number of eggs that failed to produce a neonate larva was recorded. After ten days, third instar larvae were removed from diet, quantified, and transferred to containers with vermiculite for transition to and completion of the pupal stage. Pupae remained for 16 days in vermiculite and were recovered with a sieve and quantified. All pupae were placed in small containers until emergence as adults, which were sexed and quantified. This procedure was replicated ten times per line, including a WT control.

To measure the fecundity, ten recently emerged pairs from each line were placed in cages with food and water. Each day the total number of oviposited eggs and deceased adult males and females was recorded, until generational overlap was attained (40 days, under artificial rearing conditions). This procedure was replicated 3 times per line, including the WT control.

#### Sexual competitiveness

Cages having a dimension of 30×30×30 cm with a small mango branch fixed in the center were used for sexual competitiveness studies. Cages were kept in the laboratory under controlled conditions (24–25°C, 50–60% RH and a light intensity of 61.77 Lux). Ten transgenic males and ten WT males at 10–12 d, and ten wild females at 16–20 d, were released within each cage [42]. The observation of copulating pairs was made between 15:00 and 19:00 o'clock, the typical time of sexual activity for *A. ludens* [42], which were then removed and placed in individual jars. At the end of the observation period, mating pair males were distinguished as transgenic or WT using epifluorescence microscopy. The number of copulations and type of males involved were recorded, with five cages evaluated for each transgenic strain.

#### Quantitative Real-Time PCR

Total RNA was isolated from adult *A. ludens* males, using TRIzol (Invitrogen). The iScript cDNA synthesis kit (BioRad) and 400 ng RNA were used for cDNA synthesis. Quantitative realtime PCR (qPCR) was performed on approximately 100 ng cDNA using the Fast SYBR Green Master Mix (Applied Biosystems) in a StepOnePlus real-time PCR machine (Applied Biosystems). PCR cycling conditions were: 95°C for 20 s; 40 cycles of 95°C for 3 s and 60°C for 30 s; 95°C for 15 s; 60°C for 60 s; ramp from 60°C to 95°C with +0.3°C/s; 95°C for 15 s. All reactions were performed on three replicates. Gene specific primers for DsRed (QDsRed_F/QDsRed_R) and *A. ludens Histone 3* (AlHis3; QAlHis3_F/QAlHis3_R) were designed using the Geneious 6 (Biomatters) software (Table S1 in File S1).

Relative accumulation of DsRed normalized against AsHis3 was calculated from the formula 2-ΔΔCt [43], where 2 is the reaction efficiency and ΔΔCt is the difference in AlHis3 Ct values between the calibrator (lowest DsRed expressing line *attP_*2-M6y) and the other samples, subtracted from the difference in DsRed Ct values between the calibrator (lowest DsRed expressing line *attP_*2-M6y) and the other samples.

### Data analysis/statistics

#### Demographic parameters

The population growth rate (**λ**) was calculated using the matrix model N_(*t+1)*_ = A*N_(*t)*_) [44] where A is the population-projection matrix and N_(*t)*_ is the stage-distribution vector. To construct the population-projection matrix A the life cycle of *A. ludens* was divided into five developmental phases: 1) egg, 2) neonate larva, 3) third instar larva, 4) pupae, and 5) reproductive adult (female), in order to build projection matrices. With the survival data for each developmental phase the transition rates (P*_i_*) from one phase to the next were calculated for all transgenic strains and WT control. The probability of permanence (S_5_) of reproductive adult females to generational overlap (40 days) was obtained from the number of females surviving. During this period, all oviposited eggs were recorded in order to calculate net fecundity per female of that age (F_5_). The stage-distribution vector is the total number of individuals at time *t* (N_(*t)*_) and is described by the number of individuals in each stage [44]). The resulting population-projection matrix A was iteratively multiplied by the stage-distribution vector N_(*t)*_, until the population reached a stable state for which the corresponding mathematical outcome is the Eigen-value of the transition matrix and the demographic meaning is the population growth rate (**λ**) [44]–[46]. Analysis of variance (ANOVA) and a Tukey-Kramer test with an alpha of 0.5 were used for analysis of the P*_i_*, S_5_, F_5_ and **λ** of the different transgenic strains and WT control, using the software JMP version 5.0.

#### Sexual competitiveness

The proportion of flies engaged in mating gives a measure of both the overall mating activity of the flies and the environmental conditions under which the mating tests were performed. According to standardized procedures, results of a test were rejected, if the mating proportion (MP) value was lower than 0.20 [47]. Mating competitiveness between males of each TTSS and wild type males for wild female mates was measured by the relative sterility index (RSI) [48]. An RSI, ranging between 0 and 1, at a value of 0.5 indicates that both males are equally competitive. The RSI data were analyzed using an ANOVA, followed by a Tukey-Kramer test (JMP 5.0). In addition, Chi-square analyses were performed on the total of matings recorded from five replicates, to determine whether significant differences exist in the number of matings between males of two strains.

## Results

### Transformation of *A. ludens*

To create transgenic target-site strains, the *pXLBacII[attP-PUbDsRed]* vector was injected in two rounds into a total of 1898 *A. ludens* embryos of the Moscafrut wild type strain, of which 490 larvae hatched (26%), 342 survived to the pupal stage (18%), and 312 G_0_ eclosed (157 males and 155 females; 16%). G_0_ adults were mated in 117 pools to virgins of which 110 were fertile. Putative G_1_ transgenic progeny were identified in 50 pools by DsRed fluorescence, yielding a transformation frequency of 45% based on fertile matings.

**Figure 1:**
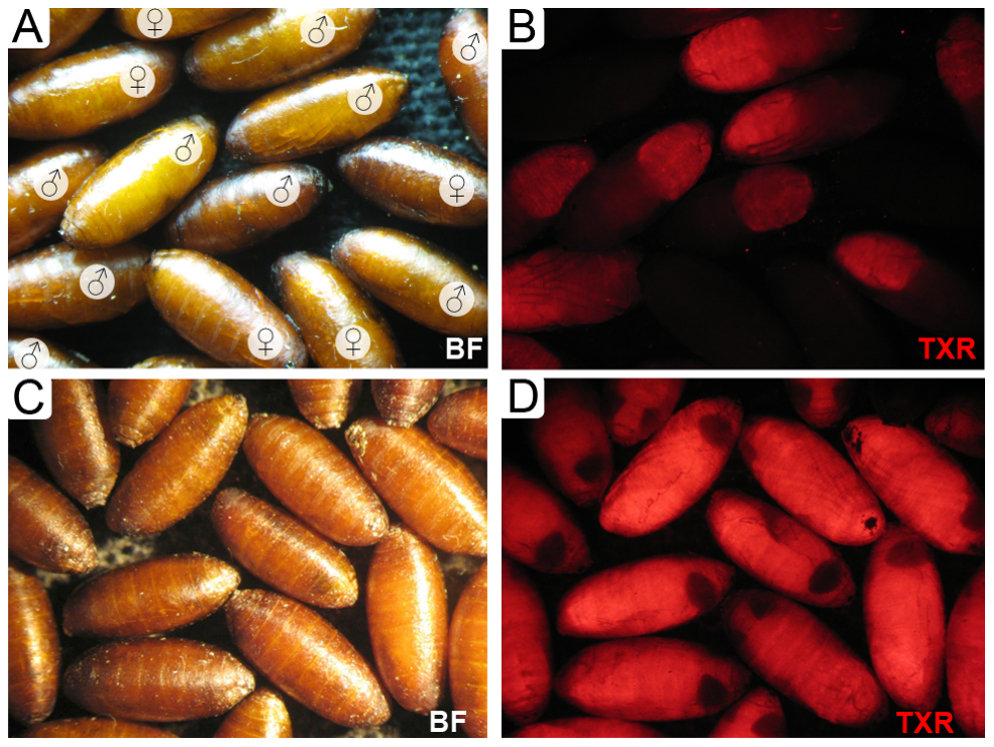
Expression of DsRed fluorescent protein in pupae. Two day old pupae of the *A. ludens* Y-linked TTSS *attP*_2-M6y (A and B) and the TTSS *attP_*F7 (C and D) are shown under brightfield (A, C) and epifluorescence optics using the TXR filter (B, D).

Transgenic males from 13 randomly selected strains were then backcrossed to wild-type Chiapas females (Table S2 in File S1). Based on Mendelian inheritance of the transgenic marker, seven of these strains (*attP*_M6, *attP*_2-M6y, *attP*_M13, *attP*_M19, *attP*_F1x, *attP*_F7, *attP*_F21) had a single integration of the transgene, with one inserted on the X chromosome (*attP*_F1x) and one inserted on the Y chromosome (*attP*_2-M6y) (Table S2 in File S1). The *attP*_M19, *attP*_F1x, and *attP*_2-M6y strains did not express fluorescence in the embryonic stage and the *attP*_F1x and *attP*_2-M6y strains did not express the marker in larvae. The strains with the strongest fluorescence based on visual inspection were *attP*_M6, *attP*_F7, and *attP*_F21. The Y-linked marker in the *attP*_2-M6y and the marker in the *attP_*F7 strain were first detected in 2d-old pupae (Fig. 1). Based on DsRed fluorescence intensity, in adults (relative to fluorescence in heterozygous adults), homozygous transgenic target-site strains *attP*_M6, *attP*_2-M6y, *attP*_M13, *attP*_M19, *attP*_F1x, *attP*_F7, and *attP*_F21 were established and subjected to fitness evaluations (Fig. S1 in File S1).

### Sequencing of target-site strain vector insertion sites

The 5′ flanking genomic DNA for several of the TTSS vector integration sites were sequenced by TAIL-PCR to verify independent integrations for each line. Five TTSS showed the canonical *piggyBac* TTAA insertion site duplications, and the distinct flanking sequences indicated independent insertion sites for these strains (Table 1). The flanking sequence for lines *attP*_M19 and *attP*_F1x could not be resolved by TAIL-PCR, but based on segregation analysis the integration site for *attP*_F1x is on the X-chromosome (Table S2 in File S1).

**Table 1:** Vector insertion site sequencing of transgenic target site strains.

Transgenic strains	5' pBac flanking sequence	BLASTn/BLASTx
*attP*_M6	…**TTAA**ATTTGTTATAGATATTTTTT…	No hit
*attP*_2-M6y	…**TTAA**AGACGCATTTATTGCTTGCA…	No hit
*attP*_M13	…**TTAA**TCTTTGCTTTTGATGTGTCC…	Heavy metal tolerance factor 1
*attP*_F7	…**TTAA**GCTAGCAGCATCTGGATCAT…	No hit
*attP*_F21	…**TTAA**TAATGAATTTCTTGAAACTT…	Tc3 like element

### Fitness evaluation of transgenic target-site strains

**Figure 2:**
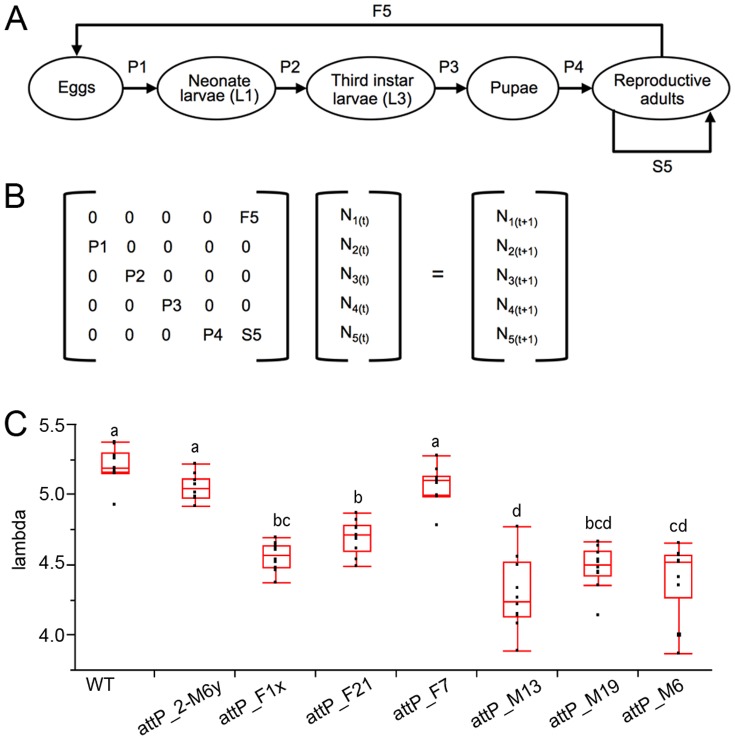
Demographic analysis. A) Diagram of the *A. ludens* stage-structured life cycle with five transition stages (arrows between nodes). P*_i_* represents the proportion of individuals in stage *i* that move to stage *i*+1 from time *t* to time *t*+1 (P_1_  =  transition frequency from eggs to neonate larvae; P_2_  =  from L1 to L3; P_3_  =  from L3 to pupae; P_4_  =  from pupae to adult), S_5_ is the proportion of individuals that remain in stage *i* from time *t* to time *t*+1, and F_5_ is the number of eggs produced per individual in stage *i* from time *t* to time *t*+1 (generational overlap); B) Stage-structured population transition matrix model. The population-projection matrix (A) is multiplied by the stage-distribution vector (N_t_) of the five elements, which represents the number of individuals in each phase at time *t*, resulting in the estimation of λ; C) Relative difference of population growth rates λ between transgenic target-site strain (TTSS) and WT strains.

First, hatching efficiency (P_1_) was monitored for all homozygous TTSS, for which significant differences were found between transgenic strains and the wild type control. The strains *attP*_F1x, *attP*_M6, and *attP*_M13 (F = 25.90; df = 7,72; P<0.001) had a mean increased mortality of 17.8% compared to wild type, while *attP*_F7 and *attP*_2-M6y performed similarly to WT. The transition from L1 to L3 (P_2_) was similar in all transgenic strains relative to WT, except for *attP*_2-M6y and *attP*_M13, which resulted in a significant 14.5% reduction in L3 larval survival compared to *attP*_F7 (F = 3.38; df = 7,79; P = 0.003). Only the *attP*_F7 strain was significantly different to WT based on pupation (P_3_) (F = 6.3237; df = 7, 79; P<0.001) and eclosion rates (P_4_) (F = 3.18; df = 7,79; P = 0.005). Nevertheless, total fecundity (F_5_) and survival of females (S_5_) of the *attP*_F7 and *attP*_2-M6y strains were similar to WT (Fig. 2 A,B; Table S3 in File S1).

All lines presented a λ-value significantly greater than 1, which is consistent for a pest species with a high growth rate such as *A. ludens*. Comparative analysis of the λ between lines revealed significant differences (F = 42.93; df = 7,72; P<0.0001): *attP*_M6, *attP*_M13, and *attP*_M19 strains were those most affected, with mean differences of 0.81, 0.91 and 0.72, respectively, relative to wild type control flies, while *attP*_F7 and *attP*_2-M6y strains did not differ significantly from WT (Fig. 2C).

#### Sexual competitiveness

Mating proportions (MP) were higher than 20% for all mating tests (mean ± SD; 67.33±9.44), indicating that the experimental conditions in the cages were suitable for mating. In general, the RSI reflected a slight tendency towards greater acceptance of wild type males by the field females in all crosses; however, the level of acceptance of transgenic males was competitive [49]. Analysis of the RSI for all cages did not show significant differences (F = 2.084; df = 5,24; P = 0.1026); however, when each strain was independently compared to wild type males, significant differences were found for *attP*_19, and *attP*_F21 (Table 2 - first analysis).

**Table 2:** Relative mating ability of TTSS and SSIS.

**First analysis**	**RSI (Mean±SD)**	**Total of mating**	***X^2^*** ** test (d.f. = 1)**	**P-Value**
Chiapas WT vs. *attP*_M6	0.37±0.076 a	22 vs 13	2.31	0.128
Chiapas WT vs. *attP*_2-M6y	0.48±0.044 a	23 vs 21	0.09	0.763
Chiapas WT vs. *attP*_M13	0.43±0.071 a	18 vs 14	0.50	0.479
Chiapas WT vs. *attP*_M19	0.33±0.071 a	24 vs 12	4.00	0.045
Chiapas WT vs. *attP*_F1x	0.45±0.137 a	20 vs 16	0.44	0.504
Chiapas WT vs *attP*_F7	0.47±0.073 a	16 vs 15	0.03	0.857
Chiapas WT vs *attP*_F21	0.34±0.087 a	21 vs 10	3.81	0.050
**Second analysis**				
Chiapas WT vs. *attP*_2-M6y	0.50±0.060 a	18 vs 18	0.00	1.00
Chiapas WT vs. *int*_2-M6y	0.51±0.056 a	18 vs 19	0.02	0.869
Chiapas WT vs *attP*_F7	0.50±0.084 a	18 vs 19	0.02	0.869
Chiapas WT vs. *int*_F7	0.27±0.050 b	31 vs 12	8.39	0.003
*attP*_2-M6y vs. *int*_2-M6y	0.47±0.038 a	18 vs 16	0.11	0.731
*attP*_F7 vs. *int*_F7	0.15±0.105 b	26 vs 5	14.22	<0.001

Letters denote the level of significant difference, with no difference among values with common letters.

Comparisons were measured between TTSS, SSIS and Chiapas wild type males based on the relative sterility index (RSI) (between cages) and on the total number of matings across all replicates (within cages).

### Site-specific integration mediated by *phiC31* integrase

**Figure 3:**
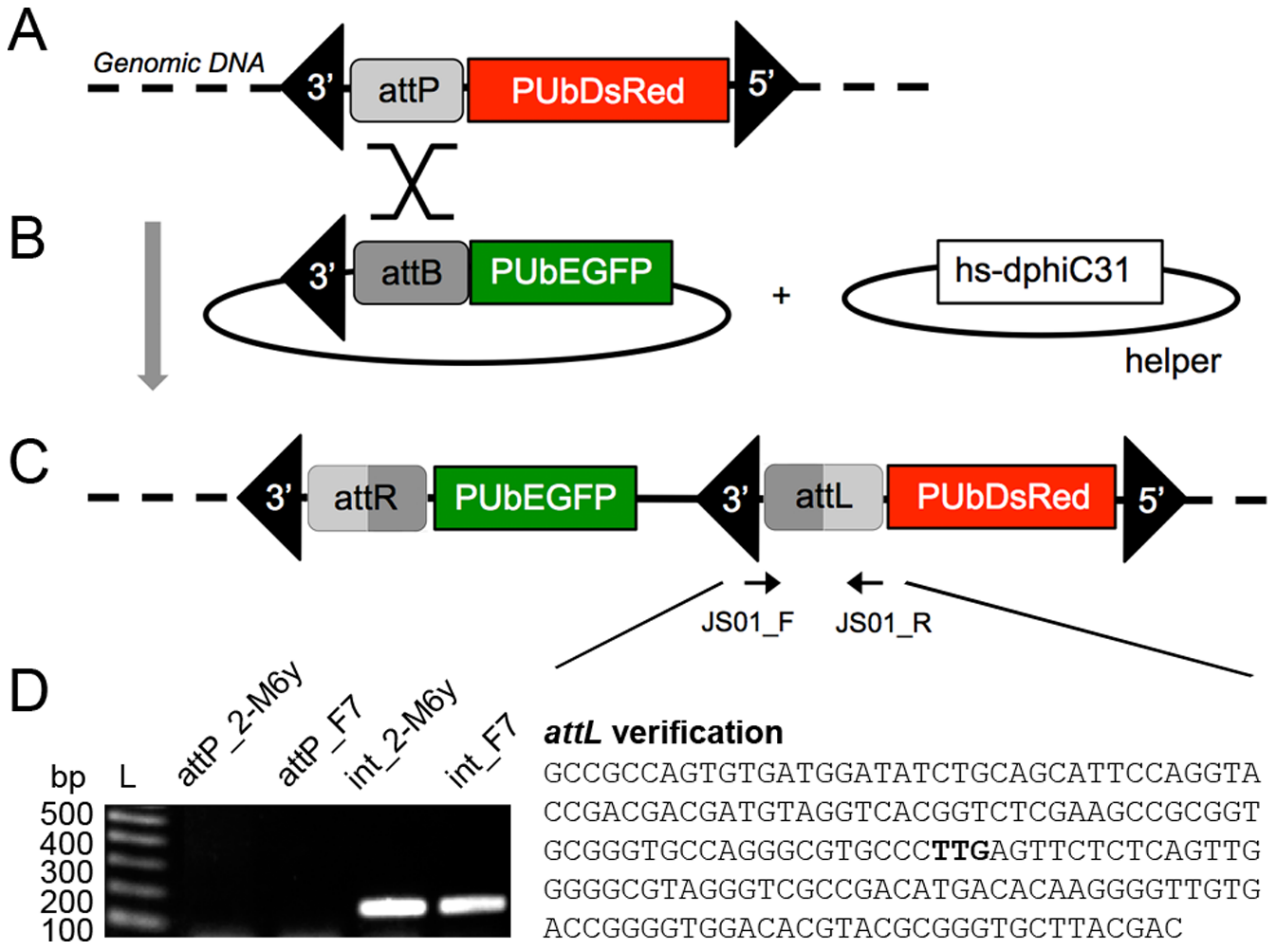
Site-specific integration in *A. ludens* mediated by *phiC31* integrase. A) Schematic of the *pXLBacII[attP-PUbDsRed]* target site vector genomic integration recombining with the *pSL_3pB-attB-PUbEGFP* donor vector, at their respective *attP* and *attB* sites, in the presence of *phs-dphiC31* integrase helper plasmid, resulting in recombinant *attR* and *attL* sites within the SSIS expressing DsRed and EGFP. B) PCR amplification products using the JS01_F and JS01_R *attL* site primers in the *attP*_2-M6y and *attP*_F7 TTSS strains and the *int*_2-M6y and *int*_F7 SSIS strains. PCR products are observed only in the SSIS lines (bottom left), having sequences corresponding to the *attL* site with the core TTG in bold (bottom right).

The strains found to be most fit by the previous evaluations, *attP*_F7 and *attP*_2-M6y, were then selected for *phiC31*-mediated site-specific integration experiments (Fig. 3). The transgene donor plasmid, *pSL_3pB-attB-PUbEGFP*, was co-injected with helper plasmid into 426 and 371 embryos of the *attP*_F7 and *attP*_2-M6y strains, respectively (Fig. 3A,B). Of these injected embryos, 95 (22.3%) and 27 (7.5%) hatched, 62 (14.6%) and 25 (6.9%) pupated, and 54 (12.7%; 26 G_0_ males and 28 G_0_ females) and 22 adults eclosed (6.1%; 10 G_0_ males and 12 G_0_ females), respectively.

All G_0_ adults (except females from the Y-linked *attP*_2-M6y strain) were individually backcrossed to wild type Chiapas virgins and G_1_ progeny was then inspected by epifluorescence microscopy for DsRed and EGFP expression. All G_1_ progeny expressed DsRed, with 8 of 42 *attP*_F7 fertile backcrosses and 2 of 10 *attP*_2-M6y backcrosses expressing EGFP, indicating a targeted integration frequency of approximately 20% (Fig. 3C). These new integrated strains were designated as *int*_F7 and *int*_2-M6y.

### Verification of site-specific integrations

**Figure 4:**
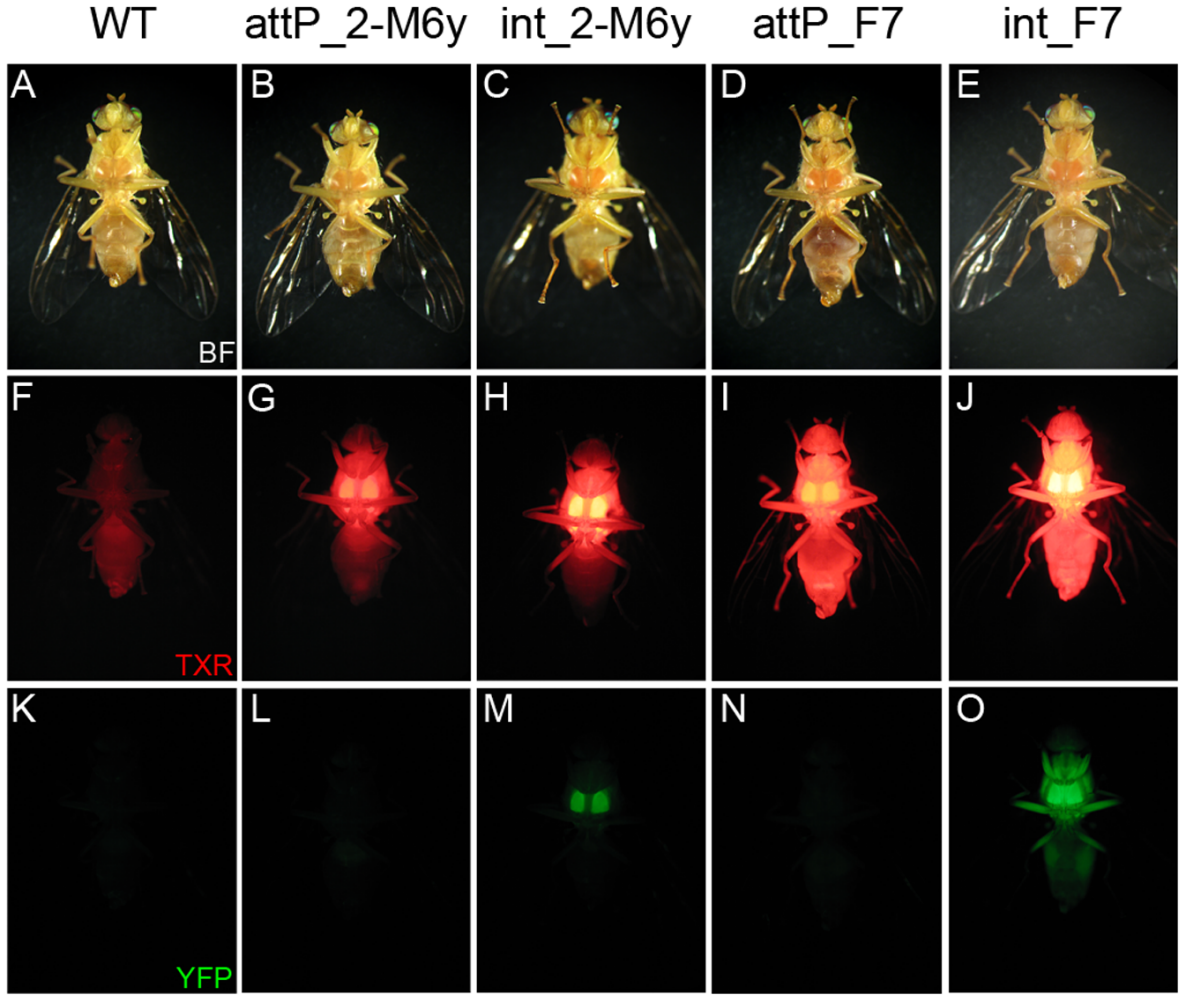
Expression of fluorescent proteins in homozygous *A. ludens* TTSS and SSIS. Images of the same individual flies from indicated lines under brightfield (BF; A-E), and epifluorescent microscopy using the Texas Red (TXR) filter for DsRed fluorescence (F-J), and the YFP filter for EGFP fluorescence (K-O). See Materials and Methods for filter specifications.

The vector *pSL_3pB-attB-PUbEGFP* was then site-specifically integrated in tandem, respective to the two marker gene orientations, in the strains *int*_F7 and *int*_2-M6y and integration was verified by PCR on *attL* sites that result from the *attP-attB* recombination event. As a negative control, the same PCR reactions were performed on the original *attP*_F7 and *attP*_2-M6y strains. PCR amplicons (181 bp) were sequenced and revealed exact identity to the expected *attL* sequence, indicating the *attB_EGFP* donor vector insertions occurred by recombination with *attP* within the expected target sites (Fig. 3D). Independent homozygous lines were then established by single pair inbreeding for successive generations with testing by segregation analysis of transformants outcrossed to WT flies and fluorescence intensity relative to heterozygous individuals. The TTSS and SSIS showed strong expression of DsRed when observed under epifluorescence microscopy (Fig. 4). In the strongest lines, including *attP*_F7, *int_*2-M6y, and *int*_F7, DsRed was detectable as red pigmentation under brightfield optics, which was not observed in WT flies (Fig. 4 C-E).

An increase in DsRed expression after successful integration of the *PUb-EGFP* marker was recognized by visual comparison of images (Fig. 4 G, H, I and J). This was confirmed by expression levels of DsRed measured by quantitative PCR, with a significant increase of DsRed in the strains *int*_2-M6y (P = 0,030) and *int*_F7 (P = 0,0001) compared to the initial target site strains (Fig. S2 in File S1).

### Fitness evaluation of target-site strains

**Figure 5:**
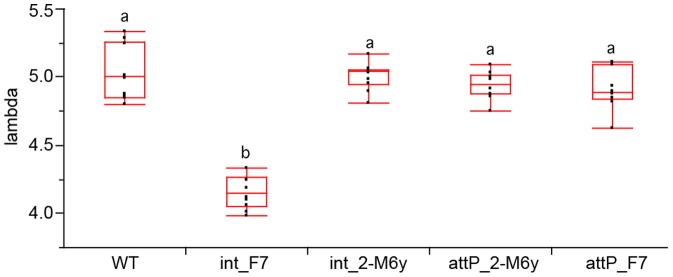
Comparison of population growth rates. Relative difference of λ between TTSS, SSIS, and WT strains is depicted.

Target-site strains with the integrated EGFP marker (*int*_2-M6y and *int*_F7) were evaluated and compared to their parental strains (*attP*_2-M6y and *attP*_F7). Most transition rates were found to vary significantly between strains (P_1_: F = 5.93; df = 4,45; P = 0.006. P_2_: F = 7.06; df = 4,45; P = 0.002. P_3_: F = 2.43; df = 4,45; P = 0.060. P_4_: F = 3.42; df = 4,45; P = 0.015), except the transition rate L3-pupa. Successful larval hatching, L1-L3 development, and female fecundity (G_5_: F = 35.38; df = 4,45; P<0.001. F_5_: F = 307.06; df = 4,45; P<0.001) was reduced in the *int*_F7 strain. In contrast to the first evaluation, the fecundity of *attP*_F7 TTS was significantly lower than wild type (Table S3 in File S1). Finally, the population growth rate of the *int*_F7 SSIS was significantly lower than the rate of all strains compared in the second analysis (F = 66.81; df = 4,45; P<0.001) (Fig. 5).

#### Sexual competitiveness

In this second analysis the MP was marginally higher than the previous test (0.76±0.153), with the RSI ANOVA analysis indicating a significant difference between experiments (F = 16.09; df = 3,19; P<0.0001). Overall, the results indicate a reduced sexual competitiveness of *int*_F7 compared to both the Chiapas WT and the parental *attP*_F7 strain. In contrast, where the level of sexual competitiveness of the *int*_2-M6y strain was comparable to the WT and *attP*_2-M6y strains (Table 2 - second analysis). Low sexual competitiveness recorded for males *int*_F7 could be due to observed flight ability problems of the strain and hence, difficulties in mating.

## Discussion

Here we describe the first creation of independent transgenic target-site strains for the Mexican fruit fly, *A. ludens*, and demonstrate the efficacy of site-specific insertion mediated by the *phiC31* system. From several TTSS, two were selected having strong transgene expression and fitness parameters to assess the impact, if any, that insertion of an additional targeted transgene might have on host strain fitness and expression of the original marker gene. One of the TTSS, *attP*_2-M6y, was not negatively impacted by the insertion of a new EGFP marker and additional sequences within the *attB* donor plasmid. However, the same insertion into the *attP*_F7 target site strain (*int*_F7) resulted in a significant negative effect on fitness.

Fitness cost evaluations of the transgenic strains were performed on the hypothesis that a change in the host insect genome caused by the integration and expression of an exogenous gene would influence the fitness of the insect [50]. To understand the effects of these changes on population statistics, we analyzed the demography of the transgenic strains. Demographic analysis was then performed to estimate the parameter lambda (λ) by using matrix models [44]. This demographic parameter provides insight into the complexity of establishing a transgenic line as a mass-reared strain useful for SIT application. In addition, detection of any detrimental effect on transgenic male competitiveness provides a predictor for the potential efficacy of a strain used in SIT.

During the first phase of this study the comparison of WT with TTSS insects in terms of transition rates, female survival to generational overlap and fecundity, reflected fitness costs in at least one stage. Except for *attP*_2-M6y and *attP*_F7, all TTSS were affected in the first transition rate life cycle (egg to larval neonate) and in fecundity. The apparent fitness cost in *attP*_2-M6y was in the transition rate of larval neonate to third instar larvae, and for *attP*_F7, in the transition rate of third instar larvae to pupae and pupae to adulthood. These fitness costs, however, did not affect the λ in either strain, which were the only strains not significantly different than wild type. This result could be explained by the strong effect of fecundity on λ, since all TTSS having λ affected suffered a significant reduction in fecundity. Similar results were found in transgenic *Aedes aegypti* where the demographic parameters of net reproductive rates, mean generation time, the intrinsic rate of natural increase and population doubling time were significantly affected [34].

The RSI for the initial sexual competitiveness comparisons did not show significant differences between any of the TTSS males and wild type males for mating effectiveness with WT females. However, all the TTSS males were less effective to varying degrees, with the most effective TTSS males coming from the *attP*_2-M6y and *attP*_F7 strains. While the RSI assessments were performed under lab conditions and not in field cages, the selective power of WT females for mates has been shown to be maintained in-lab, allowing them to discriminate males with deficiencies in their sexual courtship display [42]. That these strains can maintain mating competitiveness similar to non-transgenic field insects is also consistent with their having, among the TTSS, the most stable population growth rate.

In the second phase of this study, the potential fitness cost of the site-specific integrations was determined by comparing fitness between the host TTSS, the SSIS and wild type controls. In comparing the 2-M6y and F7 strains, only the Y-linked 2-M6y strains did not exhibit a differential effect on fitness between the original TTSS, the SSIS and WT in terms of population growth rate and sexual competitiveness. This may be explained, in part, by two factors. First, the male-specific Y-linked transgene would limit effects on population growth to only half of the population, though not on potential effects on male mating competitiveness. Secondly, the mexfly Y chromosome is thought to be highly repetitive and degenerate with few coding regions, similar to medfly and *Drosophila* [51]. Thus, potential targets effecting either fitness parameter may be relatively limited. In comparison, for the autosomal *int*_F7 SSIS, most developmental transition rates (except 3rd instar larvae to pupa), female survival to generational overlap, fecundity, population growth rate and sexual competitiveness were negatively affected compared to the host *attP*_F7 TTSS. Given that any fitness effects caused by a genomic insertional mutation should have been apparent in the *attP*_F7 TTSS, the significant effects of the secondary internal site-specific integration are not necessarily obvious. One possibility is the relatively strong over-expression of both the TTSS DsRed and SSIS EGFP markers that may have a detrimental effect together, not realized by the DsRed alone. This strong expression of exogenous genes may produce physiological effects as it accumulates in tissues [32], [52]. An interesting phenomenon was the additional expression of DsRed in both integration lines after insertion of the PUbnlsEGFP marker. Both markers are driven by the *D. melanogaster polyubiquitin* promoter and this additional PUb promoter, in tandem orientation with PUb-DsRed, could have an enhancer effect resulting in the 210-fold increase of DsRed transcripts in *int_*F7 relative to the *attP*_F7 strain, possibly contributing to the reduced fitness of *int_*F7.

We have shown that the *phiC31* system can be successfully used to create transgenic strains with a strong potential for future incorporation into SIT population control programs [50]. In this study we constructed transgenic target-site mexfly strains, at least one of which (*attP*_2-M6y) was determined to be highly fit and sexually competitive, and remained so after subsequent site-specific integration of an additional EGFP-marked transgene. The presence of the *phiC31 attP* recombination site provides a target for new transgene constructs that will allow the creation of new and more efficacious transgenic strains for pest management programs. For example, the sex-specific expression of a fluorescent marker may allow automated sex separation as larvae [53] for male-only releases, or adding a chemical–resistance gene that will allow released male survival in areas with chemical control. Given that the *phiC31* recombination system is not restricted by transgene insert size, as are transposon-based vectors [54], large multi-functional constructs can now be integrated into known genomic insertion sites in the Mexican fruit fly that should not be subjected to the potential negative effects of random genomic integrations. As shown here, the *attP_*2-M6y site supports strong transgene expression, and neither the initial transposon vector insertion nor a subsequent targeted insertion had a negative effect on transgene expression or host strain fitness. While this Y-linked TTSS will be particularly useful for specifically manipulating males, it will not be useful for bi-sexual or female-specific manipulations, though it is very likely that more suitable autosomal TTSS will be generated. However, the negative impact of a targeted insertion on *attP_F7* host strain fitness provides a critical insight that additional targeted transgene sequences are not necessarily without effect on the host strain. Given that potential insertional mutational effects on fitness should have been revealed in the TTSS strain, it is most likely that the *int_F7* strain was negatively impacted physiologically by the *attB_EGFP* donor vector. Therefore unintended physiological effects of donor vector insertions and exchanges must be considered.

## Supporting Information

File S1 **File containing Figures S1 and S2, and Tables S1-S3.**
(DOCX)
