# Supplementary material for: Fitness Cost Implications of PhiC31-Mediated Site-Specific Integrations in Target-Site Strains of the Mexican Fruit Fly, Anastrepha ludens (Diptera: Tephritidae)
Source: PLoS One. 2014 Oct 10;9(10):e109690. doi: 10.1371/journal.pone.0109690 (PMC4193812; doi:10.1371/journal.pone.0109690)

**Table S1. Oligo sequences shown in 5´ to 3´ orientation**

| L1-5pB | CATTTTGACTCACGCGGTCGTTATAGTTC |
| --- | --- |
| L2-5pB | CAGTGACACTTACCGCATTGACAAGCA |
| L3-5pB | CGACTGAGATGTCCTAAATGCACAG |
| R1-3pB | ACCTCGATATACAGACCGATAAAACACATGC |
| R2-3pB | GTCAATTTTACGCATGATTATCTTTAACGT |
| R3-3pB | CGTACGTCACAATATGATTATCTTTCTAGG |
| JS01_R | GTCGTAAGCACCCGCGTACGTGTC |
| JS01_F | GCCGCCAGTGTGATGGATATCTGC |
| P817 | CTGACTAATAAGTATAATTTGTTTC |
| P818 | ACGCATCGGGTTGATATCCGCAAATCGACGAAAATGTGTCG |
| P819 | TTTCTTGTTATAGATATCATGGACACGTATGCCGGTGC |
| P820 | ATACTTATTAGTCAGCTAGGCCGCTACGTCTTCG |
| QAlHis3_F | ATCAGTTTTGAAATCTTGAGCGAT |
| QAlHis3_R | TACTAAGCAGACTGCTCGTAAATC |
| QDsRed_F | CAAGTGGGAGCGCGTGATGAA |
| QDsRed_R | CCGTCGGAGGGGAAGTTCACG |

**Table S2.** **Phenotype segregation analysis of TTSSs**. Progeny from backcrosses of one G_1_ transgenic male to three WT Moscafrut females were screened and sorted by sex and marker expression. Significant difference is achieved if P < 0.05.

| TTSS | Transformed flies | |  | Wild type flies | | Total | *Chi*-square  (1:1) | P-value |
| --- | --- | --- | --- | --- | --- | --- | --- | --- |
|  | male | female |  | male | female |  |  |  |
| *attP*_M1 | 44 | 32 |  | 25 | 20 | 121 | 7.94 | 0.005 |
| *attP*_M3 | 38 | 42 |  | 24 | 19 | 123 | 11.13 | 0.001 |
| *attP*_M6 | 28 | 29 |  | 30 | 26 | 113 | 0.008 | 0.925 |
| *attP*_2-M6y | 87 | 0 |  | 0 | 81 | 168 | ---- | ---- |
| *attP*_M12 | 41 | 38 |  | 21 | 20 | 120 | 12.03 | 0.001 |
| *attP*_M13 | 30 | 33 |  | 34 | 32 | 129 | 0.069 | 0.795 |
| *attP*_M19 | 31 | 24 |  | 36 | 35 | 126 | 2.03 | 0.154 |
| *attP*_M21 | 43 | 39 |  | 16 | 12 | 110 | 26.50 | <0.001 |
| *attP*_F1 | 28 | 31 |  | 6 | 8 | 73 | 27.73 | <0.001 |
| *attP*_F1x | 0 | 68 |  | 60 | 0 | 128 | --- |  |
| *attP*_F7 | 49 | 45 |  | 46 | 49 | 189 | 0.008 | 0.942 |
| *attP*_F20 | 64 | 67 |  | 35 | 31 | 197 | 21.44 | <0.001 |
| *attP*_F21 | 38 | 36 |  | 35 | 32 | 141 | 0.34 | 0.556 |

**Table S3.** **Life cycle analysis of TTSS.** Average transition frequencies (P*i*), permanence of females to the generational overlap (S5), and mean net fecundity per female (F5) were calculated. Numbers are based on an initial cohort of 100 eggs (Mean±SD; P=0.05, n=10). The first analysis compared TTSS to WT, the second analysis compared WT strains to TTSS and SSIS.

| **First analysis** | **P_1_** | **P_2_** | **P_3_** | **P_4_** | **S_5_** | **F_5_** |
| --- | --- | --- | --- | --- | --- | --- |
| WT (Chiapas) | 0.92±0.01 a* | 0.81±0.10 ab | 0.97±0.02 a | 0.95±0.05 a | 0.80±0.10 a | 4091 ± 22 a |
| *attP*_M6 | 0.72±0.05 c | 0.74±0.18 ab | 0.97±0.02 a | 0.91±0.05 ab | 0.70±0.10 a | 2613 ± 17 b |
| *attP*_2-M6y | 0.91±0.02 a | 0.68±0.07 b | 0.98±0.02 a | 0.92±0.03 a | 0.70±0.10 a | 3902 ± 23 a |
| *attP*_M13 | 0.76±0.06 bc | 0.65±0.16 b | 0.99±0.01 a | 0.89±0.06 ab | 0.70±0.10 a | 2540 ± 45 b |
| *attP*_M19 | 0.80±0.04 b | 0.82±0.09 ab | 0.97±0.02 a | 0.90±0.11 ab | 0.73±0.11 a | 2342 ± 19 b |
| *attP*_F1x | 0.76±0.06 bc | 0.76±0.08 ab | 0.97±0.02 a | 0.91±0.04 ab | 0.66±0.05 a | 2944 ± 19 b |
| *attP*_F7 | 0.92±0.03 a | 0.85±0.09 a | 0.93±0.03 b | 0.83±0.05 b | 0.80± 0.00 a | 4132 ± 23 a |
| *attP*_F21 | 0.81±0.06 b | 0.75±0.11 ab | 0.97±0.02 a | 0.94±0.05 a | 0.76±0.05 a | 3034 ± 13 b |
| **Second analysis** | | | | | | |
| WT (Chiapas) | 0.93±0.02 ab | 0.79±0.10 a | 0.98±0.03 a | 0.93±0.03 ab | 0.82±0.04 a | 3613 ± 157 a |
| *attP*_2-M6y | 0.91±0.02 abc | 0.72±0.04 ab | 0.98±0.01 a | 0.93±0.02 ab | 0.80±0.04 a | 3603 ± 121 a |
| *int*_2-M6y | 0.95±0.01 a | 0.82±0.07 a | 0.99±0.01 a | 0.90±0.04 b | 0.76±0.04 a | 3539 ± 166 ab |
| *attP*_F7 | 0.89±0.05 bc | 0.74±0.09 a | 0.98±0.01 a | 0.96±0.03 a | 0.80±0.04 a | 3415 ± 74 b |
| *int*_F7 | 0.87±0.05 c | 0.63±0.09 b | 0.95±0.02 a | 0.94±0.03 ab | 0.62±0.04 c | 1885 ± 157 c |

*Letters denote the level of significant difference, with no difference among values with common letters

**Figure S1. Comparison of heterozygous and homozygous females from the *A. ludens* transgenic target site strain *attP*_F7.** Adult females of *attP*_F7 are shown under brightfield (A) and epifluorescence using the TXR filter (B). The heterozygous female (left) shows a weaker DsRed intensity compared to the homozygous female (right). Heterozygous and homozygous conditions were verified by segregation analysis through backcrosses to WT.


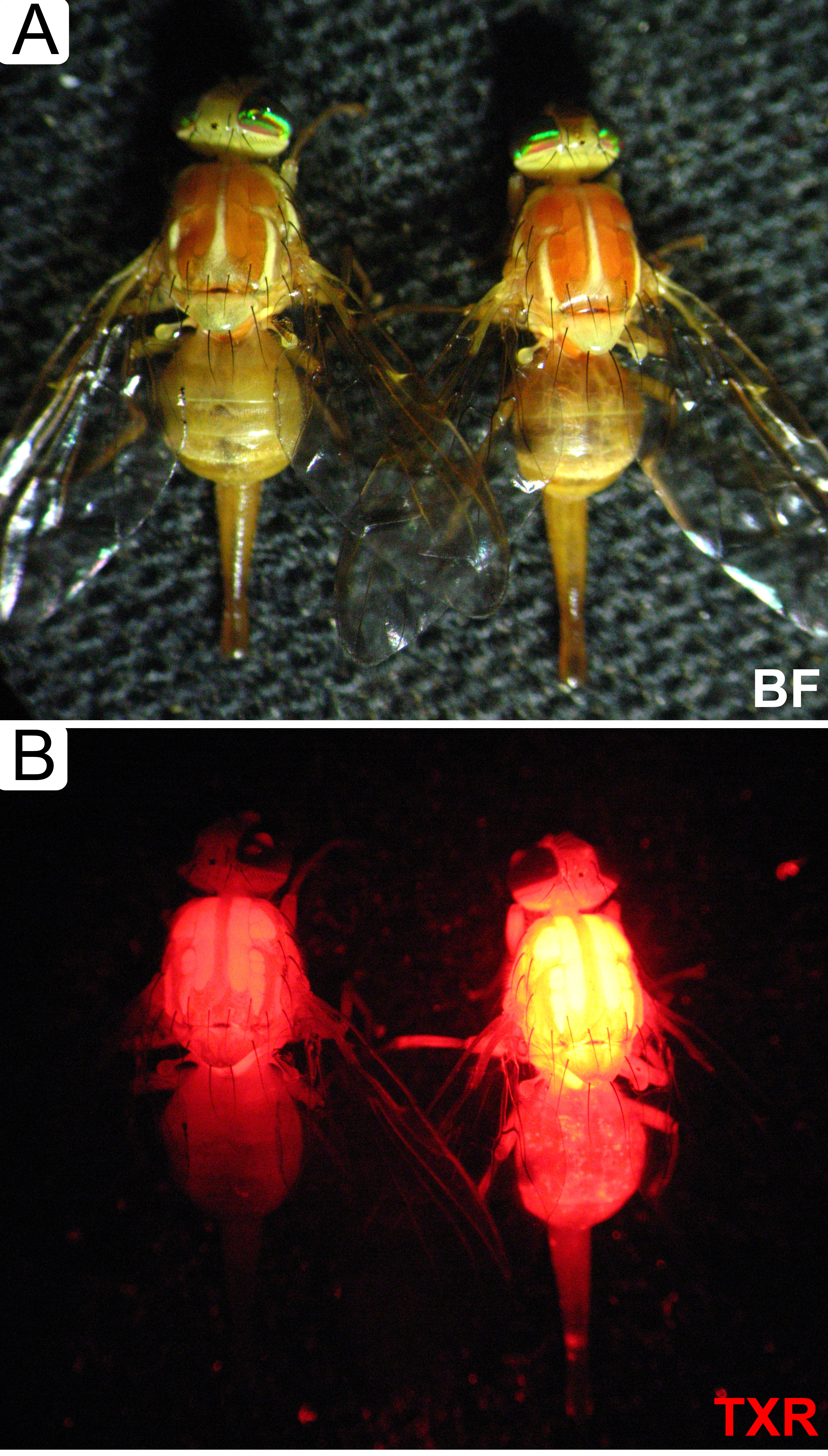


**Figure S2. Relative expression of DsRed transcript in *A. ludens* strains.** DsRed expression was compared by quantitative PCR on cDNA generated from adult flies. Relative accumulation of DsRed transcript normalized against *AlHis3* transcript using the strain *attP_2-M6y* as a calibrator is shown. Error bars show the S.E., with the mean fold change from three independent experiments shown above.


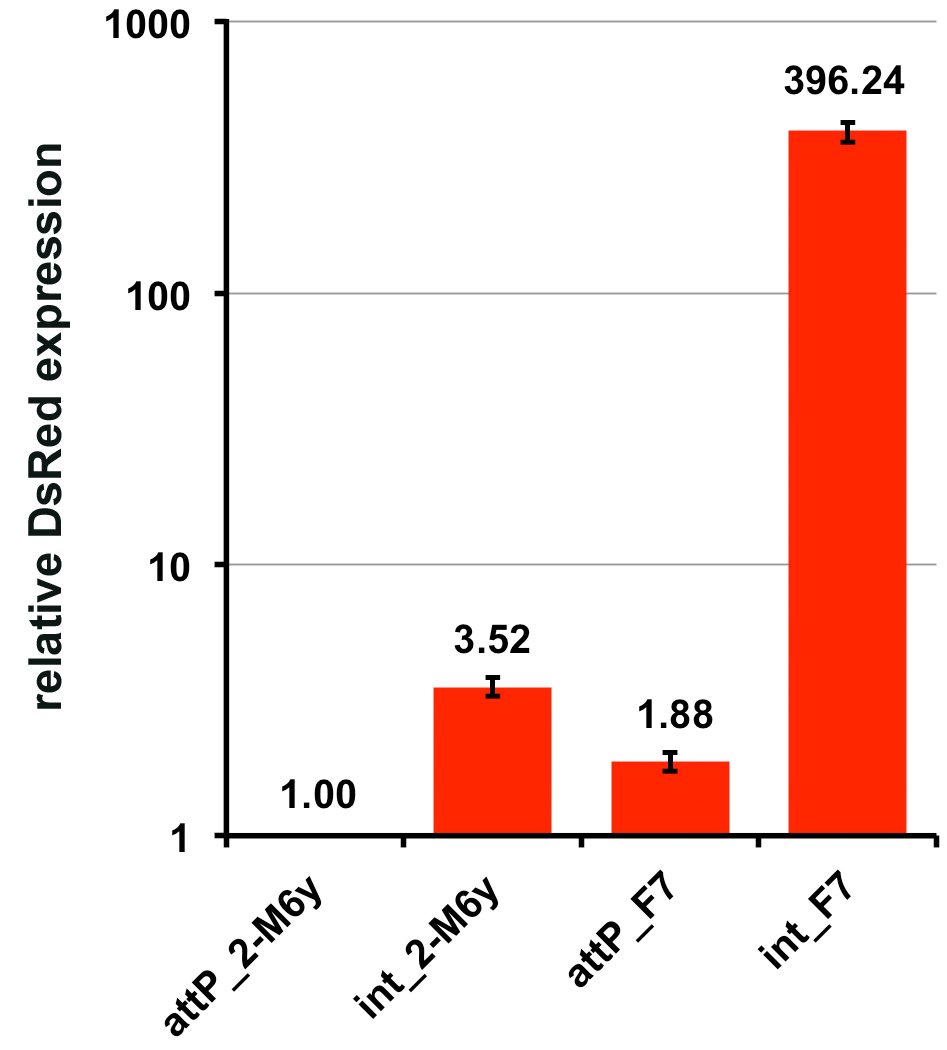

Supplement: File S1 — File containing Figures S1 and S2, and Tables S1-S3. (DOCX) [file pone.0109690.s001.docx]
